# A Prospective Assessment of Near-Infrared Image-Guided Sentinel Lymph Node Identification in Esophageal and Esophagogastric Junction Cancer

**DOI:** 10.3390/cancers18162534

**Published:** 2026-08-07

**Authors:** Daniela Molena, Tamar Nobel, Marisa Sewell, Amirthavarsini Manikandan, Kay See Tan, Matthew J. Bott, Katherine D. Gray, Hans Gerdes, Pari Shah, Makoto Nishimura, Laura Tang, Bernard J. Park, Smita Sihag, David R. Jones

**Affiliations:** 1 Thoracic Service, Department of Surgery, Memorial Sloan Kettering Cancer Center, 1275 York Avenue, New York, NY 10065, USA; 2 Department of Epidemiology and Biostatistics, Memorial Sloan Kettering Cancer Center, New York, NY 10065, USA; 3 Gastroenterology, Hepatology, and Nutrition Service, Department of Medicine, Memorial Sloan Kettering Cancer Center, New York, NY 10065, USA; 4 Department of Pathology and Laboratory Medicine, Memorial Sloan Kettering Cancer Center, New York, NY 10065, USA

**Keywords:** esophageal cancer, sentinel lymph node, lymph node mapping

## Abstract

**Objective:** To evaluate the feasibility of sentinel lymph node (SLN) identification in distal esophageal and esophagogastric junction (EGJ) adenocarcinoma and determine whether the SLN concept applies to this disease. **Methods:** Patients undergoing esophagectomy for clinical stage I-IVA esophageal and EGJ adenocarcinoma underwent SLN mapping by near-infrared detection of indocyanine green (ICG) injected via endoscopy before esophagectomy. Lymphatic drainage patterns were recorded, and the first identified nodes (SLNs) were harvested. Standard esophagectomy and lymphadenectomy were performed. SLN status was compared with overall nodal status. **Results:** Of 90 included patients, 77 received ICG; SLN mapping was successful in 64 (83%). Ten patients had two SLN stations identified; the remainder had one. The most common SLN location was along the left gastric artery (36/74 [48%]), followed by the paracardial (17/74 [23%]) and periesophageal (15/74 [20%]) stations. Seven patients had SLNs positive for metastasis (in one, the SLN was the only positive node; in the rest, metastatic nodes were identified in the same or other stations). Of the 57 patients with negative SLNs, 11 (19%) had ≥1 positive node in the same or different nodal station. The false-negative rate was 61.1%. **Conclusions:** SLN mapping with ICG is feasible in patients with distal esophageal and EGJ adenocarcinoma, with a high rate of successful node identification. Occult nodal metastasis in patients with negative SLNs limits the reliability of SLN assessment as a determinant for selective lymphadenectomy.

## Simple Summary

This study evaluated whether surgeons could identify the first lymph node likely to contain cancer (“sentinel lymph node”) in patients with lower esophageal and gastroesophageal junction cancers. Using a fluorescent dye during surgery, sentinel lymph nodes were successfully identified in most patients. However, many patients whose sentinel nodes appeared cancer-free still had cancer in other lymph nodes. Because of this high false-negative rate, sentinel lymph node mapping was not reliable enough to guide limited lymph node removal. The findings suggest that while the technique is feasible, standard lymph node removal during esophagectomy remains important to avoid missing cancer.

## 1. Introduction

Nodal metastasis is one of the most important factors that determines the prognosis for patients with esophageal adenocarcinoma [1,2]. Extensive lymphadenectomy at the time of esophagectomy improves staging, decreases rates of local recurrence, and prolongs survival [3].

The lymphatic spread of esophageal cancer is multidirectional and includes regional nodal stations in the neck, abdomen, and chest [4]. Although the celiac trunk is the most common site of nodal metastasis in distal esophageal and esophagogastric junction (EGJ) adenocarcinoma, mediastinal involvement has been observed in up to 25% of patients, and multiple nodal stations are often involved concurrently [5,6]. Moreover, tumor location does not reliably predict the distribution of nodal metastases, and, therefore, extensive (two-field) lymphadenectomy at the time of esophagectomy is the standard of care in the US [7,8].

Previous efforts to limit the extent of lymphadenectomy by the use of sentinel lymph node (SLN) assessment for prediction of nodal disease have not established that the SLN concept can be reliably applied to esophageal cancer [9]. However, recent advances and interest in endoscopic therapy and esophagus-preserving approaches have prompted a renewed focus on sentinel node assessment in esophageal cancer [10]. Although the current evidence on this topic is limited to that from feasibility studies, centers around the world have started to use sentinel node assessment to inform the treatment of patients with early-stage esophageal cancer [11,12].

The SLN concept is based on the premise that the first draining lymph node(s) from a primary tumor accurately reflects the overall nodal status [13]. This concept has been validated for and widely adopted in melanoma and breast cancer, but it remains unproven for esophageal cancer [14,15].

We sought to assess the patterns of lymphatic drainage of distal esophageal and EGJ adenocarcinoma and to determine whether the SLN concept applies to this disease.

## 2. Materials and Methods

### 2.1. Study Design and Patients

We performed a prospective single-arm study from 2020 to 2024 to determine the effectiveness of near-infrared (NIR) imaging for lymphatic mapping and assessment of SLNs in patients with esophageal or EGJ adenocarcinoma. This study was approved by the institutional review board at Memorial Sloan Kettering Cancer Center (IRB#20-201), and all patients gave informed consent. Eligible patients were aged ≥18 years, had pathologically confirmed adenocarcinoma of the distal third of the esophagus or EGJ, and were scheduled to undergo minimally invasive esophagectomy for clinical stage I-IVA disease.

### 2.2. Surgical Procedure

During the laparoscopic phase of the procedure, after all trocars were placed, the surgeon injected 1 mL of indocyanine green (ICG) doubly diluted in sterile water (0.25 mg/mL) into the esophageal submucosa at the superior and inferior edges of the tumor (0.5 mL per injection) by use of a standard adult endoscope with an endoscopic injection needle (Video S1). An NIR-capable camera (5 mm or 10 mm, 45-degree; PINPOINT, Novadaq Technologies, Bonita Springs, FL, USA, or PINPOINT Endoscopic Fluorescence Imaging System, Stryker, San Jose, CA, USA) was used to capture real-time images for lymphatic mapping and SLN identification.

After perilesional injection of the ICG solution, the surface of the esophagus was carefully examined for evidence of (1) fluorescence in the region of the parenchymal injection, (2) lymphatic migration, and (3) NIR-positive lymphatic pathways and lymph nodes (Video S2). Any NIR-positive SLNs identified by the surgeon were removed and sent to Pathology as separately labeled lymph node specimens, distinct from the remainder of the lymph nodes removed. In situ real-time back-table NIR imaging was performed on all removed specimens to confirm resection of the lesion in question and to characterize the area of NIR labeling. The incidence of identified malignant disease was compared between the NIR-identified SLNs and all other lymph nodes sampled or included in the lymphadenectomy specimen. All patients then underwent complete lymphadenectomy and esophagectomy in accordance with institutional standards, as previously described [16]. The operating surgeon determined the specific operative and postoperative management of the patients in accordance with institutional protocols.

### 2.3. Pathologic Evaluation of SLNs

**Figure 1:**
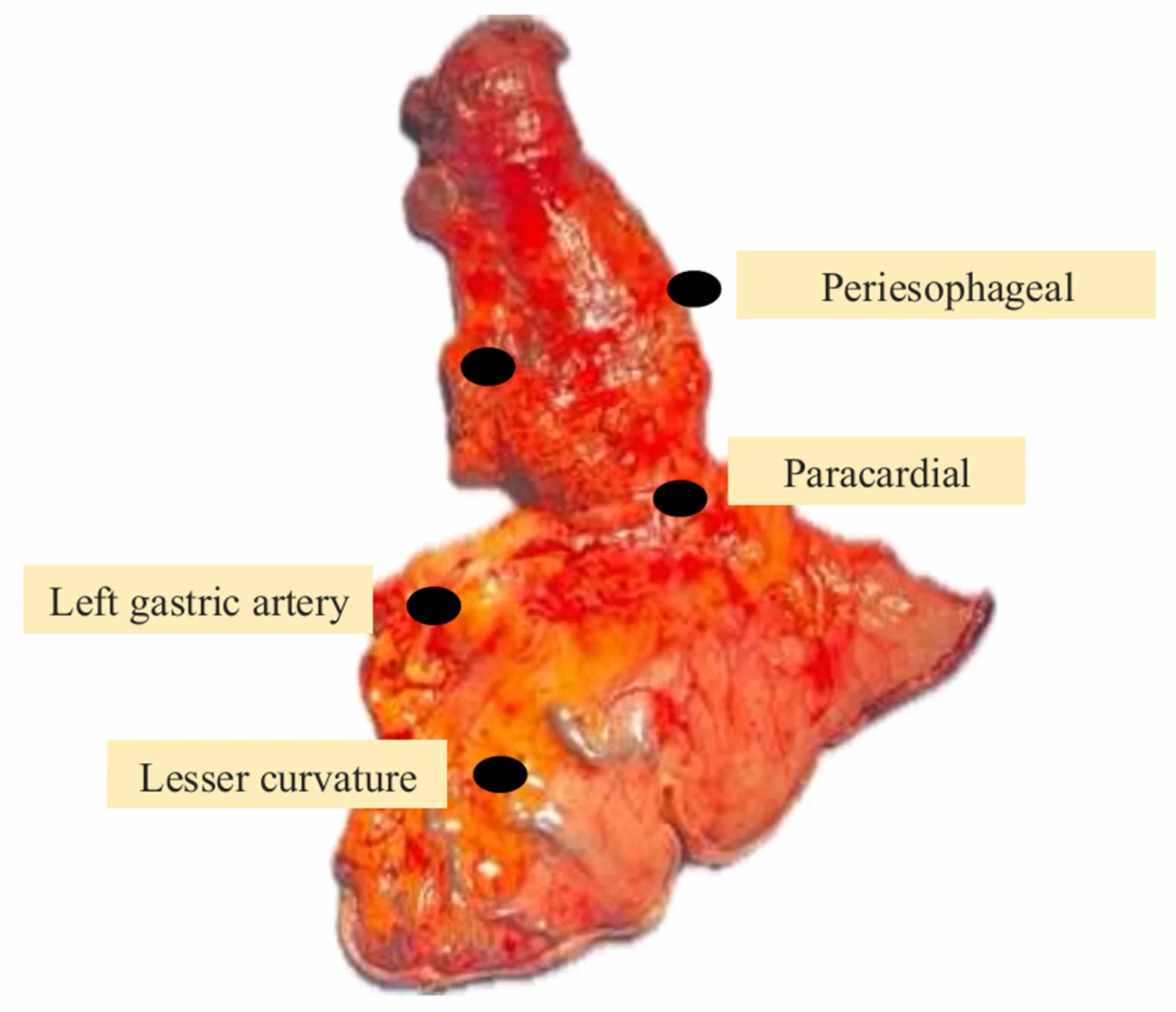
Gross esophageal specimen denoting the location of lymph nodes. Lymph nodes sent as separate specimens to the pathologist, including the hepatic artery, splenic artery, and infracarinal nodes, are not pictured.

All of the lymph nodes detected via NIR imaging and submitted to Pathology as sentinel node(s) were evaluated by use of permanent hematoxylin and eosin-stained sections using standard clinical protocols. An SLN was considered to be positive if it had a tumor deposit of any size within the lymph node, including isolated tumor cells. The esophagectomy specimen was sectioned in accordance with a standardized protocol; each nodal station was labeled appropriately by the surgeon at the time of resection or during grossing of the specimen (Figure 1). Small lymph nodes were submitted as whole and larger lymph nodes were sectioned according to their size. Sentinel lymph nodes were sent as separate pathologic specimens.

### 2.4. Outcomes and Analysis

Relevant clinicopathologic characteristics were summarized descriptively as frequencies and percentages of the median (interquartile ranges [IQRs]). Surgical data included the number of lymph nodes identified and removed (both NIR-fluorescent and NIR-nonfluorescent nodes), the visualization of specific lymphatic pathways to those nodes, the timing of injection and node identification, and the anatomic location and distribution of both NIR-fluorescent and NIR-nonfluorescent lymph nodes. Failure of SLN mapping was defined as a lack of evidence of NIR-fluorescent lymph nodes.

Patients who had successful SLN mapping were considered to be evaluable for assessment of whether the SLN pathologic status was reflective of the overall nodal status. The false-negative rate of NIR-guided SLN biopsy in patients with esophageal cancer was evaluated. The false-negative rate (the proportion of confirmed node-positive cases in which the SLN biopsy was negative) was considered as 1 minus sensitivity. The final pathologic status of “sentinel nodes” was established and was considered the reference standard for the assessment of the accuracy of SLN identification using NIR-guided imaging. By standard clinical practice, pathologic diagnosis (also known as the “reference standard”) included the SLN status when the SLN was positive. Therefore, if the SLN was positive, the pathologic diagnosis was “node positive”—consequently, in this context, the concept of “false-positive” did not apply.

Identification of any metastasis in the SLN was considered to be an event (i.e., SLN-based diagnosis was positive). On the basis of SLN biopsy and final pathologic results, the false-negative rate of SLN biopsy (the proportion of confirmed node-positive cases in which the SLN biopsy was negative) was determined: false-negative cases/(true-positive cases + false-negative cases).

With the assumption of a true false-negative rate of 10% (for all patients with any stage of disease), we estimated that a sample size of 30 patients with lymph node metastasis would allow us to estimate the false-negative rate of NIR-guided mapping with a margin of error (95% confidence interval [CI] half widths) of ±10.7%. We report the sensitivity and positive and negative predictive values along with 95% CIs.

## 3. Results

### 3.1. Demographic Characteristics

**Figure 2:**
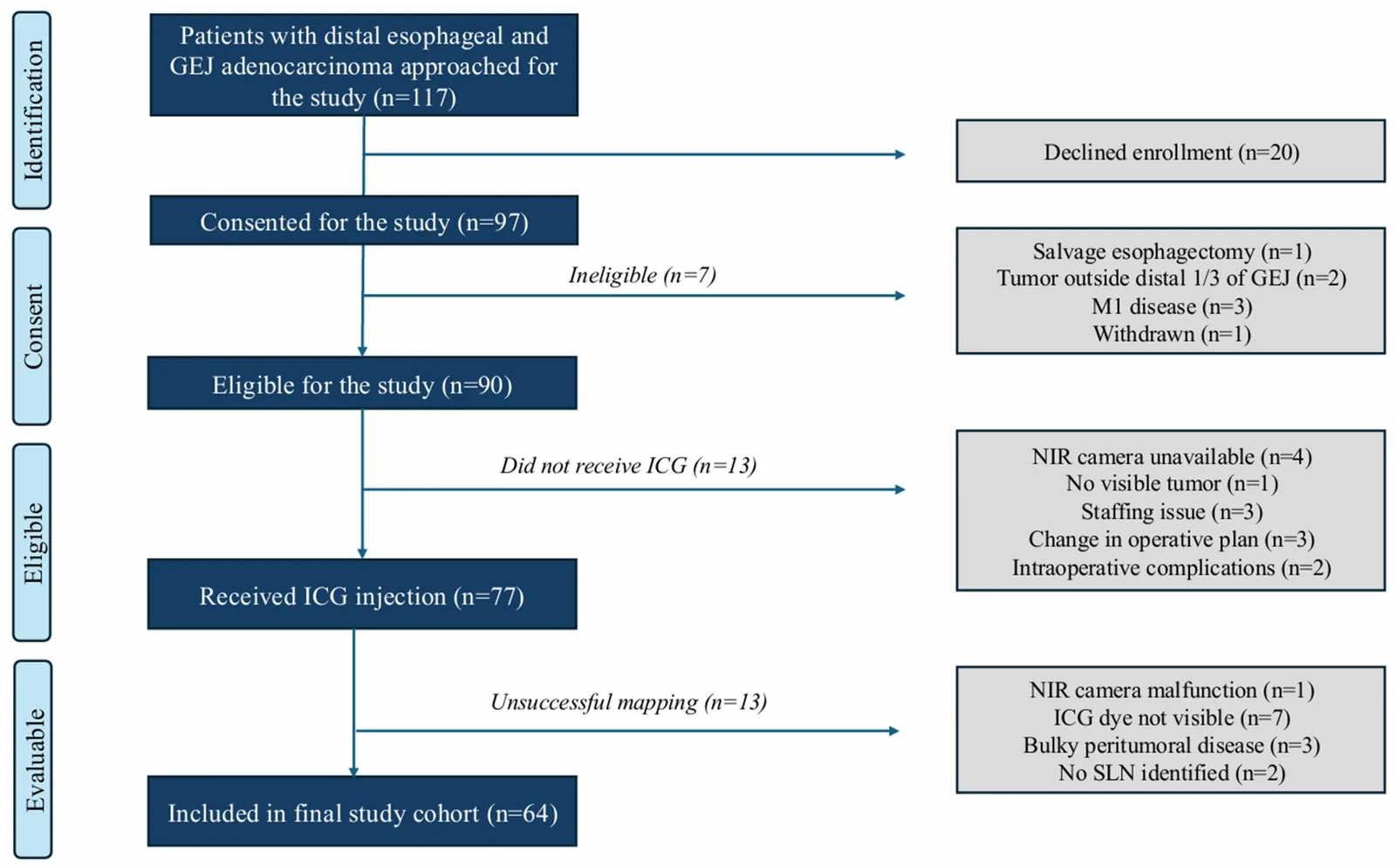
CONSORT diagram.

Ninety eligible patients were enrolled in the study; however, only 77 underwent endoscopic injection of ICG. The most common reason that eligible and enrolled patients did not undergo injection of ICG was an issue with the NIR-capable camera (Figure 2). Successful SLN mapping was achieved in 64 of the 77 patients who underwent ICG injection (83.1%; 95% CI, 72.8–90.6%), corresponding to an overall mapping success rate of 71.1% (64/90; 95% CI, 60.6–80.1%) among all enrolled patients. The reasons for failed SLN mapping in patients who had successful injection of ICG are detailed in Figure 2. The 64 patients with successful SLN mapping were evaluated as the study cohort. The median age was 66 years (IQR, 63–71), most patients were men, and most patients had an Eastern Cooperative Oncology Group status of 1 (Table 1). Most patients (n = 36 [56%]) had clinical stage III disease. Seventeen patients (26%) were treatment-naive. The remaining 47 patients (75%) underwent neoadjuvant therapy, including 32 who underwent chemoradiation therapy.

### 3.2. SLN Mapping

Overall, among evaluable patients, endoscopic injection of ICG was completed in a median of 3 min (IQR, 3–7). ICG was then visualized. The median time from the first injection to the first visualization of the dye was 10 min (IQR, 7–15). Migration of ICG to the lymph node(s) was recorded as nodal drainage mapping. An SLN was identified in all evaluable patients. Most patients had only one identifiable SLN station, although 10 patients had two SLN stations identified. The most common SLN station was along the left gastric artery (36/74 [48%]), followed by the paracardial (17/74 [23%]) and periesophageal (15/74 [20%]) stations.

The median number of lymph nodes identified in SLN stations per patient was 1 (range, 1–5). The median number of lymph nodes removed in the final pathologic specimen was 34 (IQR, 25–42). Overall, 18 patients had positive nodes and 46 patients had negative nodes on final pathologic evaluation. Seven patients had a positive SLN on final pathologic evaluation. In one of these patients, the SLN was the only positive node; in the other six, additional positive nodes were found in other stations (Table 2). Of the 57 patients with negative SLNs, 11 (19%) had ≥1 positive node in the same nodal station (four patients) or different nodal stations (seven patients) (Table 2). In all four patients with a negative SLN in the same station where nodal metastasis was identified on final pathologic evaluation, the SLN was along the left gastric artery.

Among patients with pathologically node-positive disease, SLN biopsy had a sensitivity of 38.9% (95% CI, 19.4–61.4%). The false-negative rate (or the proportion of patients who had pathologic node-positive disease with a negative SLN biopsy) was 61.1% (95% CI, 38.6–80.8%). The positive predictive value was 100% (95% CI, 59.0–100%), and the negative predictive value was 80.7% (95% CI, 68.6–89.6%).

### 3.3. Treatment-Naive Patients

Overall, 17 of 64 evaluable patients were treatment-naive. The majority of these patients were men, and the median age of these patients was 64 years (IQR, 60–70) (Table 1). Most of these patients had clinical stage I disease (13/17 [76%]). Only one treatment-naive patient had pathologic node-positive disease. Additionally, in this patient, the SLN was the only positive node. Of the 16 treatment-naive patients with negative SLNs, none had pathologic node-positive disease on final pathologic evaluation.

## 4. Discussion

Our results confirm the feasibility of SLN mapping by use of endoscopic ICG injection in patients with distal esophageal and EGJ adenocarcinoma, with a rate of successful SLN mapping of 83%. However, the presence of occult nodal metastasis in patients with histologically negative SLNs raises concerns about the reliability of SLN assessment as the sole determinant for performing selective lymphadenectomy. In this cohort, a substantial proportion of node-positive patients would have been understaged if SLN status alone had been used to guide surgical resection.

There is growing interest in esophagus-preserving techniques for patients with T1 disease. However, evidence suggests that, for patients with high-risk disease, such as those with submucosal invasion >500 um, lymphovascular invasion, or poorly differentiated tumors, complete endoscopic resection is insufficient because of the elevated risk of nodal metastases in these patients [18,19,20]. Of the patients in our cohort who had early-stage disease and were treatment-naive, the one patient with pathologic node-positive disease had a positive SLN; however, the extremely small number of treatment-naive patients limits any meaningful inference regarding the predictive value of SLN assessment in this subgroup. These findings should be interpreted as hypothesis-generating only. Neoadjuvant therapy, which was administered in the majority of patients in this study, may also alter lymphatic drainage patterns and tracer migration, potentially influencing SLN identification and accuracy. Accordingly, any differences in lymphatic mapping performance between treatment-naïve and neoadjuvant-treated patients could not be meaningfully assessed in this study. Future larger studies are needed to determine whether SLN assessment may contribute to risk stratification in early-stage disease and potentially aid in identifying appropriate candidates for esophagus-preserving therapy.

Although lymphatic mapping has been successfully implemented for other malignancies, the validity and feasibility of this approach for esophageal cancer remains a matter of debate. Traditional methods of nodal mapping (blue dye, radiocolloid lymphoscintigraphy) have proven to be more challenging in esophageal surgery than in other settings [21,22]. For example, a study by Boone et al. demonstrated that intraoperative lymphatic drainage was identified by use of a gamma probe in only 38% of patients [23]. The high rate of failure was attributed to the observed accumulation of radioactive tracer in the periesophageal nodes in direct proximity to the tumor, resulting in tumor radioactivity overshadowing that of adjacent lymph nodes. Similarly, in a study that assessed blue dye-guided dissection, Burian et al. noted limited success attributable to poor visualization of blue-stained nodes, which were either obscured until the mediastinal pleura was opened or blackened as a result of anthracosis [9]. In addition, radioactive tracer and dual-tracer methods carry the risk of patient exposure to radiation and require specialized equipment for intraoperative detection [24]. ICG fluorescence-guided sentinel node biopsy has been successfully used for several types of cancer. In a recent meta-analysis that included 513 patients across eight different types of cancer, the rate of detection was as high as 96%, with 86% sensitivity and 100% specificity [25]. We previously demonstrated that intraoperative NIR imaging with ICG is a safe and feasible method for mapping lymphatic drainage during esophagectomy [11,26,27].

In a pilot study from our group that included nine patients with esophageal or EGJ adenocarcinoma, ICG was injected into the esophageal submucosa endoscopically before the laparoscopic phase of the esophagectomy [11]. There were no perioperative deaths, and no procedure-related adverse events were observed. In eight of nine patients, lymphatic drainage was first visualized along the left gastric nodes. Three patients had nodal metastases; in all three of these patients, the first ICG-identified station corresponded to histologically positive nodes. In contrast, patients with histologically negative SLNs identified by NIR had no additional nodal metastases in the lymphadenectomy specimen. In addition to these findings, Hachey et al. reported the successful identification of metastatic nodes by use of NIR imaging in six of nine patients with locoregional esophageal adenocarcinoma [26]. Of note, NIR-positive nodes had ICG uptake ex vivo.

In another study, which included 20 patients with superficial esophageal cancer, the authors demonstrated that intraoperative NIR imaging detected SLNs in 95% of cases, with one false-negative result in a node with bulky metastatic disease [27]. Jimenez-Lillo et al. reported a systematic review and meta-analysis that examined the use of ICG for sentinel node detection in patients with esophageal cancer. They reported high sensitivity but low specificity for the rate of nodal detection. Moreover, the included studies had a low number of patients, which contributed to a wide prediction interval and uncertainty regarding the use of the technique with sufficient reliability [28]. Nevertheless, European centers are performing sentinel node mapping for high-risk T1 esophageal adenocarcinoma—not only to select patients for esophagectomy but also to select resection of metastatic nodes as a definitive approach with the omission of esophagectomy [12,29]. This appears to be a risky choice for patients with early-stage disease, which would likely be curable by means of esophagectomy.

Our study has several limitations. This is a single-arm prospective study of patients from a single high-volume cancer center, which may limit the generalizability of our findings. We did not evaluate potential learning-curve effects. Although increasing experience may influence technical success and mapping performance, the relatively small sample size precludes meaningful temporal subgroup analysis. As such, the potential impact of operator experience on SLN identification cannot be excluded. Additionally, exclusion of 13 enrolled patients due to technical or procedural factors may introduce attrition bias. These exclusions were related to limitations of the mapping technique itself, including imaging failure, lack of ICG visualization, bulky disease, and inability to identify a sentinel lymph node, and therefore reflect real-world feasibility constraints rather than selective exclusion. Nevertheless, these cases highlight an important limitation of the current technique and should be considered when interpreting the reported mapping success rate. Additionally, the majority of these patients received neoadjuvant therapy, which may limit the applicability of these results to patients with early-stage disease who are treatment-naive. The relatively small number of patients with pathologically node-positive disease (n = 18) limits the sensitivity and false-negative rate estimates, and the corresponding confidence intervals should be interpreted accordingly. These findings should therefore be considered exploratory and hypothesis-generating. Larger multicenter studies are needed to more precisely define the performance characteristics of SLN mapping in esophageal adenocarcinoma. However, our results are strengthened by the expertise that is characteristic of a high-volume cancer center, and each pathologic specimen was evaluated by an expert gastrointestinal pathologist.

## 5. Conclusions

NIR SLN mapping by use of endoscopic peritumoral injection of ICG is a feasible and safe technique for the identification of draining lymph nodes in patients with esophageal cancer. SLN mapping may have a role in improving understanding of lymphatic drainage patterns and informing future risk stratification strategies. However, our data do not support its use to guide organ-preserving esophageal cancer surgery, or the use of the sentinel lymph node concept in esophageal cancer outside of clinical trials. Large, multicenter cohorts are needed, particularly in treatment-naïve patients with early-stage disease, before the SLN concept can safely be applied to esophageal adenocarcinoma patients and to select the use of esophagus-preserving techniques.

## Figures and Tables

**Table 1:** Demographic characteristics of evaluable patients.

Characteristic	Overall (n = 64)	Neoadjuvant (n = 47)	Treatment-Naive (n = 17)
Sex			
Male	53 (83)	39 (83)	14 (83)
Female	11 (17)	8 (17)	3 (12)
Age, years	66 (63–71)	67 (64–72)	64 (60–70)
Race			
White	59 (92)	44 (94)	15 (88)
Asian	3 (5)	3 (6)	0 (0)
Other	2 (3)	0 (0)	2 (12)
Comorbidities			
Cardiac	42 (66)	29 (62)	13 (81)
Pulmonary	6 (9)	4 (9)	2 (12)
Clinical stage			
I	13 (20)	0 (0)	13 (76)
II	6 (9)	2 (4)	4 (24)
III	36 (56)	36 (77)	0 (0)
IVA	9 (14)	9 (19)	0 (0)
Neoadjuvant therapy		47 (100)	0
Chemoradiation		32 (67)	0
Procedure			
Ivor Lewis	63 (98)	47 (100)	16 (94)
3-Hole	1 (2)	0 (0)	1 (6)
Minimally invasive surgery	63 (98)	47 (100)	17 (100)
Lymphovascular invasion	14 (22)	10 (21)	4 (24)
Positive margins	2 (3)	2 (4)	0 (0)
Differentiation			
Well	2 (3)	0 (0)	2 (12)
Moderate	27 (42)	17 (36)	10 (59)
Poor	35 (55)	30 (64)	5 (29)
Pathologic T stage			
0	15 (23)	15 (32)	0 (0)
1a	7 (11)	2 (4)	5 (29)
1b	15 (23)	7 (15)	8 (47)
2	12 (19)	8 (17)	4 (24)
3	15 (23)	15 (32)	0 (0)
Pathologic N stage			
0	46 (72)	30 (64)	16 (94)
1	8 (13)	7 (15)	1 (6)
2	6 (9)	6 (13)	0 (0)
3	4 (6)	4 (9)	0 (0)
Total lymph nodes removed	34 (25–42)	35 (24–44)	31 (25–40)

Data are no. (%) or median (interquartile range).

**Table 2:** Location of SLNs, SLN metastases (if present), and all resected LNs in patients with pathologic LN metastasis.

Total SLNs	Positive SLNs	SLN Location (No. of Positive LNs)	Total LNs	Positive LNs	Location of Pathologically Positive Nodes (No. of Positive LNs)
3	1	Paracardial (1)	44	1	Paracardial sentinel (1)
1	1	Left gastric artery (1)	25	2	Left gastric sentinel (1), left gastric (1)
5	2	Paracardial (0), left gastric (2)	39	3	Left gastric sentinel (2), left gastric (1)
4	2	Left gastric artery (1), paracardial (1)	25	5	Left gastric sentinel (1), paracardial sentinel (1), level 7 (1), paraesophageal (2)
1	1	Periesophageal (1)	40	9	Periesophageal sentinel (1), paraesophageal (6), paracardial (2)
1	1	Lesser curve (1)	23	13	Lesser curve sentinel (1), paracardial (2), lesser curve (6), left gastric (4)
5	3	Left gastric artery (3)	49	26	Left gastric sentinel (3), periesophageal (8), paracardial (4), lesser curve (3), left gastric (8)
1	0	Paracardial (0)	21	1	Left gastric (1)
1	0	Gastrohepatic ligament (0)	15	1	Paracardial (1)
1	0	Periesophageal (0)	43	1	Splenic artery (1)
1	0	Cardia (0)	31	1	Paracardial (1)
4	0	Left gastric artery (0)	47	1	Periesophageal (1)
1	0	Paracardial (0)	37	1	Left gastric (1)
1	0	Periesophageal (0)	40	3	Tracheoesophageal groove (2), level 2R (1)
1	0	Left gastric artery (0)	27	3	Paracardial (1), left gastric (2)
3	0	Left gastric artery (0)	44	3	Periesophageal (1), left gastric (2)
1	0	Left gastric artery (0)	29	4	Paracardial (3), left gastric (1)
1	0	Left gastric artery (0)	22	11	Periesophageal (1), paracardial (4), left gastric (5), level 7 (1)

Each row represents 1 patient. Lymph node stations are according to the American Joint Committee on Cancer 8th edition [17].

## Data Availability

Data unavailable due to patient privacy.

## Supplementary Materials

The following supporting information can be downloaded at: https://www.mdpi.com/article/10.3390/cancers18162534/s1. Table S1: Characteristics of patients with a false-negative sentinel lymph node; Video S1: Injection of ICG into the esophageal submucosa; Video S2: Examination for fluorescence.

## Abbreviations

The following abbreviations are used in this manuscript:

CRT	Chemoradiation Therapy
EGJ	Esophagogastric Junction
ICG	Indocyanine Green
IQR	Interquartile Range
IRB	Institutional Review Board
LN	Lymph Node
NIR	Near-Infrared
SLN	Sentinel Lymph Node
US	United States
