# Supplementary material for: A Prospective Assessment of Near-Infrared Image-Guided Sentinel Lymph Node Identification in Esophageal and Esophagogastric Junction Cancer
Source: Cancers (Basel). 2026 Aug 7;18(16):2534. doi: 10.3390/cancers18162534 (PMC13510751; doi:10.3390/cancers18162534)
Supplement: Supplementary file 1 [file cancers-18-02534-s001.zip › supp table S1.pdf]

Supplementary Table S1. Characteristics of false negative patients.

**Characteristic**	**False Negatives (N=11)**
N	11
Clinical Stage	
I	0
II	2
III	6
IVA	3
Procedure	
Minimally invasive surgery	11
Ivor Lewis	11
Lymphovascular invasion	4
Positive margins	1
Differentiation	
Well	0
Moderate	3
Poor	8
Pathologic Stage	
I	0
II	0
IIIA	6
IIIB	7
IVA	1
